# A laboratory platform for studying rotational dust flows in a plasma crystal irradiated by a 10 keV electron beam

**DOI:** 10.1038/s41598-023-28152-8

**Published:** 2023-01-18

**Authors:** D. Ticoş, E. Constantin, M. L. Mitu, A. Scurtu, C. M. Ticoş

**Affiliations:** National Institute for Lasers, Plasma and Radiation Physics, 077125 Bucharest, Romania

**Keywords:** Plasma physics, Experimental particle physics, Fluid dynamics, Physics, Nonlinear phenomena

## Abstract

A novel laboratory platform has been designed and built for the irradiation of a plasma crystal (PC) with an electron beam (e-beam) having an energy around 10 keV and a current of tens of milliamperes. The pulsed e-beam collimated to a few millimeter-size spot is aimed at a crystal made of dust particles levitated in a radio-frequency (RF) plasma. The platform consists of three vacuum chambers connected in-line, each with different utility: one for generating free electrons in a pulsed hollow-anode Penning discharge, another for the extraction and acceleration of electrons at $\sim 10$ kV and for focusing the e-beam in the magnetic field of a pair of circular coils, and the last one for producing PCs above a RF-driven electrode. The main challenge is to obtain both a stable e-beam and PC by insuring appropriate gas pressures, given that the e-beam is formed in high vacuum ($\lesssim 10^{-4}$ Torr), while the PC is produced at much higher pressures ($\gtrsim 10^{-1}$ Torr). The main diagnostics include a high speed camera, a Faraday cup and a Langmuir probe. Two applications concerned with the creation of a pair of dust flow vortices and the rotation of a PC by the drag force of the e-beam acting on the strongly coupled dust particles are presented. The dust flow can become turbulent as demonstrated by the energy spectrum, featuring vortices at different space scales.

## Introduction

Plasma crystals (PCs) are collections of charged microparticles (or dust) immersed in a low temperature plasma disposed periodically in both horizontal and vertical planes^1–5^. In a typical lab setup the dust particles levitate in the plasma sheath of a horizontal electrode, where the electric force acting on them is proportional with the sheath field and is opposed to the force of gravity. A stable plasma crystal is however obtained when the repulsive screened Coulomb dust-dust forces, the friction force of the gas (or drag exerted by the neutral atoms), the ion drag force and a confining force which keeps the dust particles together are all in equilibrium^6,7^. Since the dust particles are negatively charged and positioned from each other at distances of the order of the plasma Debye length, they are strongly coupled^8^.

The plasma crystal can be subjected to external forces such as those generated by electric and magnetic fields^9–12^, centrifugal forces^13^, plasma jets^14^, laser beams^15–17^, injected charged particle beams^18–21^ or combinations of some of these forces, e.g. laser and magnetic field^22^. In all of these cases the complex dynamics of the dust particles within the crystal leads to the observation of interesting physical phenomena such as the dust acoustic or the longitudinal dust lattice waves^23^, solid to liquid phase transitions^5,17,24^, shear induced dust flows^16^, secondary emission^25^, field emission^26^, hypercharging of the dust particles^18,19,27^, dust vortices^20^, and rotation of the dust structure^28–30^.

In this work we present a novel irradiation technique of PCs with an electron beam (e-beam) which allows us to study the interaction of energetic electrons with strongly-coupled dust particles immersed in plasma. The acceleration voltage of the electrons in the beam can be varied in the range $\sim 8$ to 14 kV, while the obtainable e-beam current set by the performance of an electron source (i.e. a hollow anode Penning discharge) is in the range $\sim$ 1–30 mA. The e-beam has a circular spot with a diameter of a few mm. The energy of the electrons in the e-beam is 4 orders of magnitude higher than the thermal energy of the electrons originating in the RF plasma (which is a few eV) where the dust crystal resides. In a RF plasma the drag force on a dust particle exerted by these low temperature plasma electrons is weak, much smaller than the electric force which levitates the dust particle and therefore can be neglected^31^. By contrast, an e-beam with energy at the level of $\sim 10$ keV can push the dust particles and accelerate them up to large terminal speeds $\sim$ 1–10 mm s$^{-1}$, inducing interesting kinetic effects^20,21^.

We present here two novel applications of e-beam irradiation of a PC. In the first we show the formation of two large symmetrical dust flow vortices induced by the e-beam inside the PC. We also demonstrate that the dust flow can become turbulent, especially at low e-beam currents. The second application is concerned with the full rotation of a PC which keeps its symmetry, irradiated sideways by the e-beam.

Plasmas generated by e-beam irradiation are of interest in several areas of applied and fundamental physics spanning from industrial processes such as semiconductor etching used in the fabrication of silicon wafers^32–36^ to laboratory and space plasmas^37–40^. The e-beam is an important tool for the manipulation of dust particles in plasma which can induce dynamics with some unique properties such as sheared, laminar and turbulent dust flows^20^. It can also help to better understand the physics of new collective phenomena observed in strongly coupled charged fluids, such as the generation of simple and multiple vortices which eventually can lead to turbulence^41–49^.

## Results

### Plasma crystal irradiation technique

**Figure 1:**
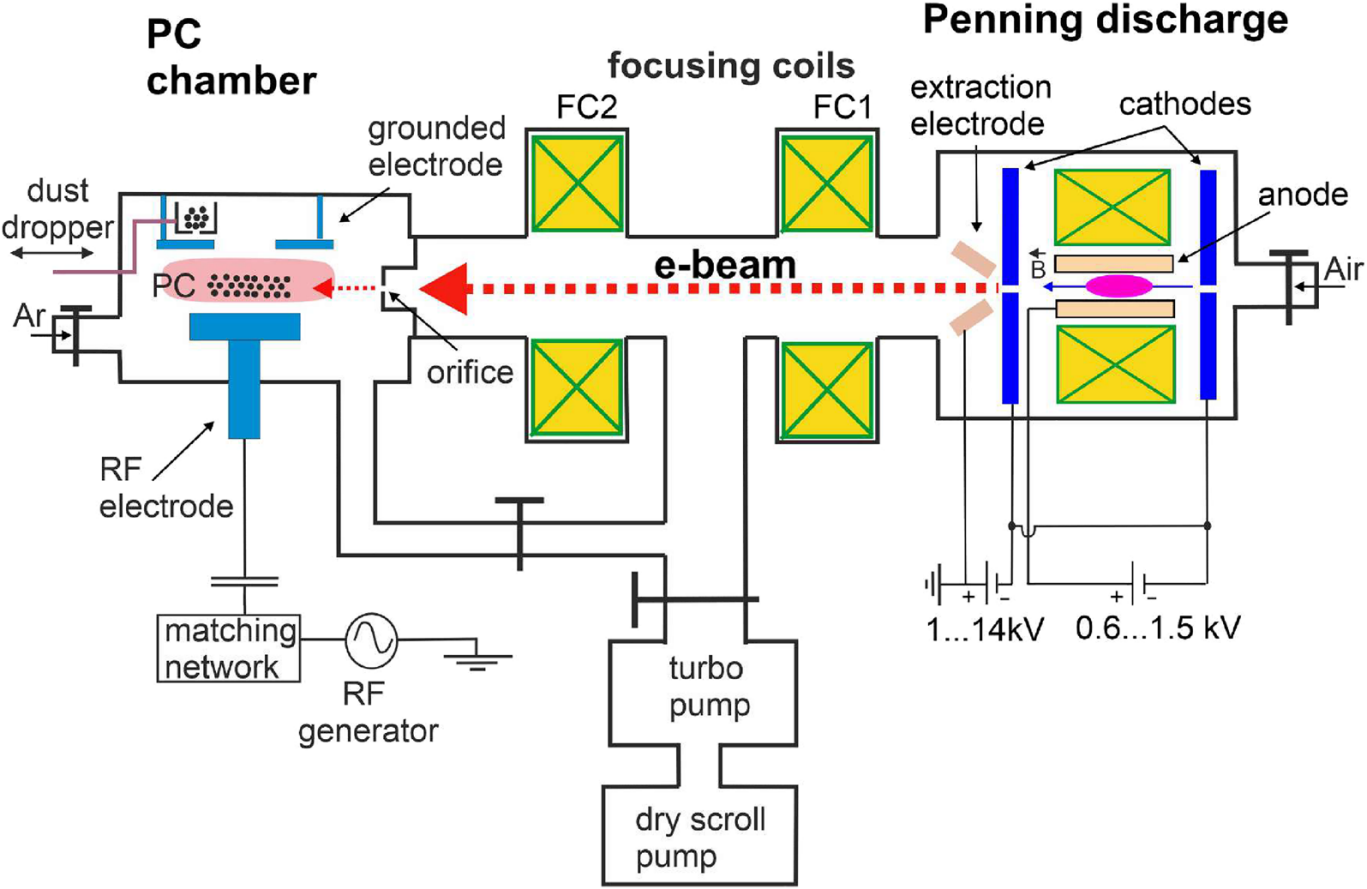
Details of the laboratory platform (not to scale): from right to left the first vacuum chamber is for producing free electrons in a pulsed Penning discharge, the second vacuum chamber (the e-beam channel) is for the formation of the e-beam and the third vacuum chamber is dedicated to the dust crystal (PC) production in a RF plasma. The free electrons extracted and accelerated from the Penning discharge are focused into a beam which is further passed through the 0.5 mm orifice into the RF plasma chamber and aimed at the levitated dust particles.

**Figure 2:**
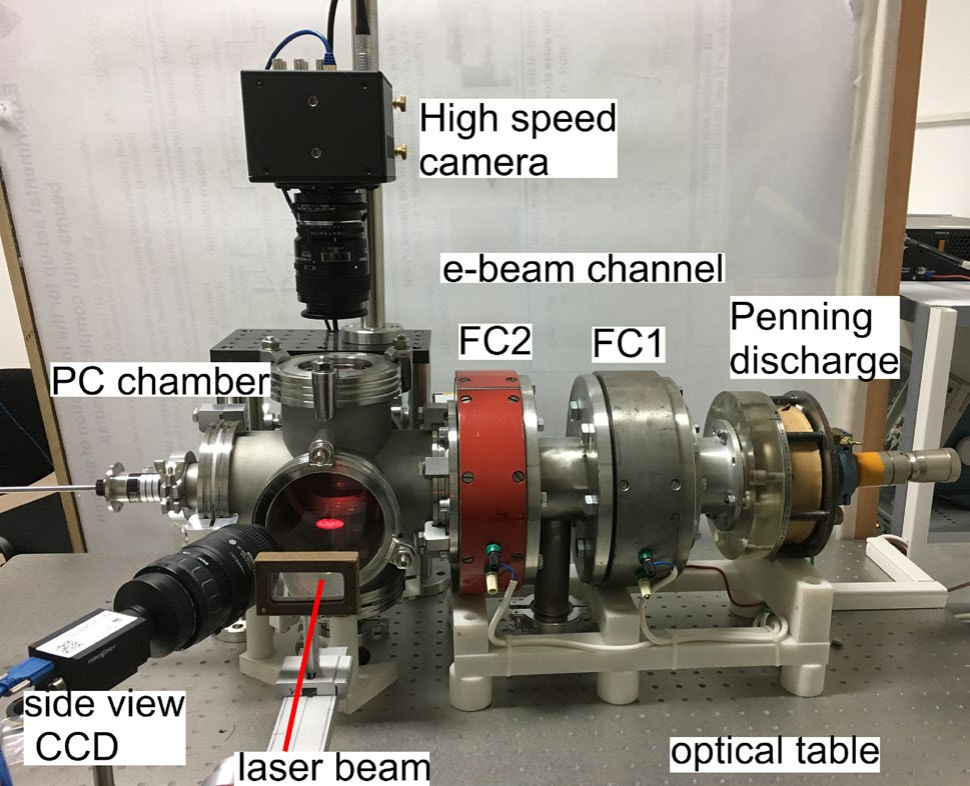
The laboratory platform for studying rotational dust flows in a PC irradiated by a $\sim 10$ keV e-beam.

A description of the PC irradiation technique is presented in Fig. 1, while the full laboratory platform is shown in the image of Fig. 2. The setup consists of three in-line connected vacuum chambers, each with a dedicated purpose. The first vacuum chamber hosts a pulsed Penning discharge where free electrons are produced. The second vacuum chamber is the beam channel where the e-beam is formed and accelerated by a high-voltage (HV) potential ($\sim 10$ kV) relative to an extraction electrode. This electrode is at the ground potential but the Penning discharge itself is biased at the HV required to accelerate the electrons^50^. The third vacuum chamber is for producing PCs in a RF discharge between two parallel plate electrodes. The e-beam is carried over and injected into this last chamber and aimed at the levitated dust particles. In the following we describe the peculiarities of each vacuum chamber and the main operational characteristics.

### The pulsed Penning discharge

**Figure 3:**
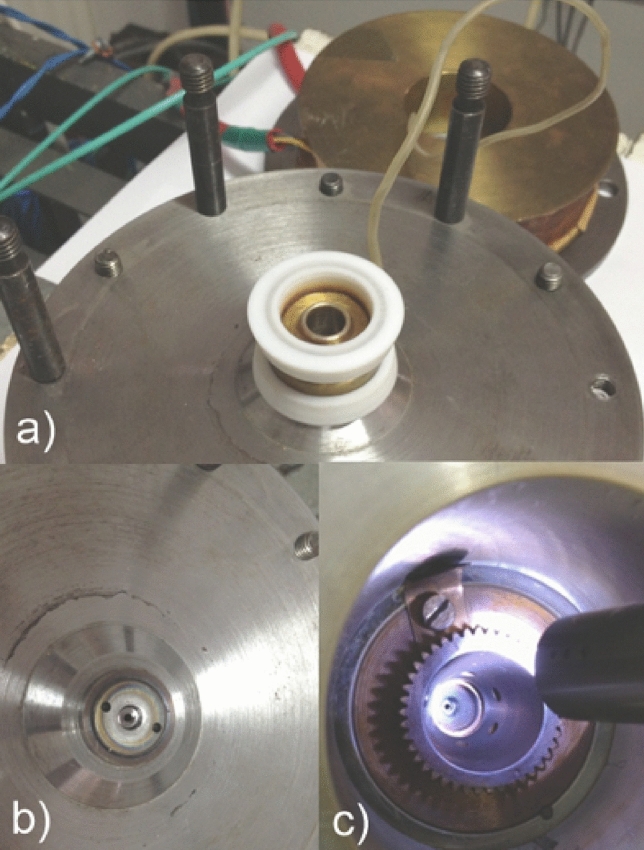
The main parts of the pulsed Penning discharge: (**a**) hollow anode made of yellow brass with teflon insulators inserted at its ends. The two cathodes are installed over these teflon rings. The coil (shown in brown) is inserted axially and contained by the fixing rods; (**b**) first cathode with 1 mm orifice for air insert, at one end of the electrode setup; (**c**) second cathode with 3 mm hole (in the center of the illuminated image) and conical toothed extracting electrode: free electrons are extracted from the Penning discharge through this hole. Electrons are accelerated between the cathode (biased at “-” polarity of the HV, e.g. − 10 kV) and the grounded toothed electrode.

The Penning discharge uses air at a pressure $\approx 10^{-3}-10^{-1}$ Torr introduced through a needle valve. Plasma is produced between two cathodes in the shape of disks and a hollow cylindrical anode inserted between them^50^. The electrode configuration is drawn in Fig. 1 while images of the unit for producing the free electrons and of the electrodes are shown in the Figs. 2 and 3, respectively.

The hollow anode is surrounded by a coil producing a magnetic field that confines the electron trajectories, shown on the side in Fig. 3a. The axial magnetic field can reach 650 Gauss at a constant current of 150 mA passing through the coil. The two cathodes are provided each with a hole, one with 1 mm in diameter used for introducing air from the needle valve and the other with a 3 mm diameter for extracting the electrons, presented in the Figs. 3b,c, respectively.

The Penning system consists of two cathodes and one anode. The cathodes are biased at the negative polarity of a HV Glasmman source (e.g. at − 10 kV). A grounded toothed ring electrode is placed at $\sim 2$ mm from the cathode in order to extract the electrons from the discharge and accelerate them, as shown in Fig. 3c. The electrons are accelerated in the electric field between the biased cathode and the grounded ring electrode which is of the order of 50 kVcm$^{-1}$.

**Figure 4:**
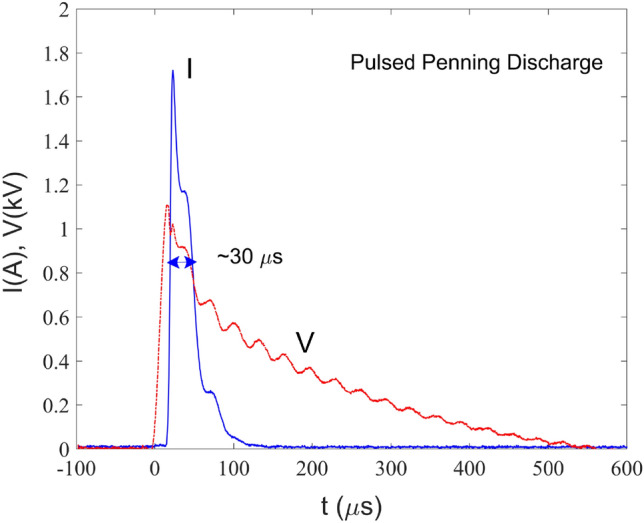
Voltage and current pulses of the Penning discharge set at a frequency of 46 Hz. The e-beam is formed during the time interval marked with the arrow.

Plasma is formed by applying a pulsed voltage with peak value between 0.6 and 1.5 kV from a generator built in-house. In Fig. 4a typical I–V characteristic of the Penning discharge is presented. Once the voltage has reached $\sim ~1$ kV the discharge is ignited and the current increases rapidly up to a peak value of 1.7 A. The voltage then drops gradually in time in about 0.5 ms, while the current falls to zero after  150 $\upmu$s. The pulse is reinstated after 21.7 ms which corresponds to the repetition frequency of 46 Hz. The FWHM duration of the pulse is $\approx 30~\upmu$s while its frequency can be changed to several preset values, between 46 and 180 Hz.

### The e-beam channel

The extracted electrons are passed into a large ($\sim 10$ cm inner diameter) tubular vacuum chamber pumped down at a base pressure of $10^{-5}$ Torr. Along this chamber two large external coils (FC1 and FC2) are installed and positioned at 20 cm from each other, measured from their centers. They each produce an axial magnetic field up to 200 Gauss. By powering these coils with currents between 1 and 5 A and at voltages of about 3–10 V, the magnetic field configuration ensures that the extracted electrons are collimated along the axis of the chamber.

At the other end the e-beam channel is coupled to the RF plasma chamber through a dedicated flange. The formed e-beam is passed into the RF plasma chamber through an orifice with 0.5 mm diameter (see Fig. 1). The flange was designed in such a way that its central part can be replaced in order to accommodate orifices with different diameters or shapes. The diameter of the e-beam in cross section before entering through this orifice is established by the focusing magnetic fields. A good focusing allows to obtain e-beam currents past the orifice with peak values up to 30 mA in the RF plasma chamber, while the whole e-beam current inside the channel can be as high as 150–200 mA.

The diameter of the orifice needs to be limited and cannot be increased too much above 0.5 mm due to the high vacuum requirement inside the e-beam channel, otherwise the gas from the RF plasma chamber would fill the channel. The pressure in the RF plasma chamber can be up to 3 orders of magnitude higher. An alternate way to keep the high vacuum is to pass the e-beam through a thin separating membrane, although this possibility has not been tested in our setup^51^. In this case the tensile strength in the membrane due to the pressure difference on both sides of it should be well evaluated. Also the stopping power of electrons in the material needs a careful assessment, otherwise the membrane could block the e-beam (e.g. the range after which an e-beam with energy 14 keV is fully attenuated in an Al sample is $\approx$ 3–4 microns).

### The RF plasma interaction chamber

**Figure 5:**
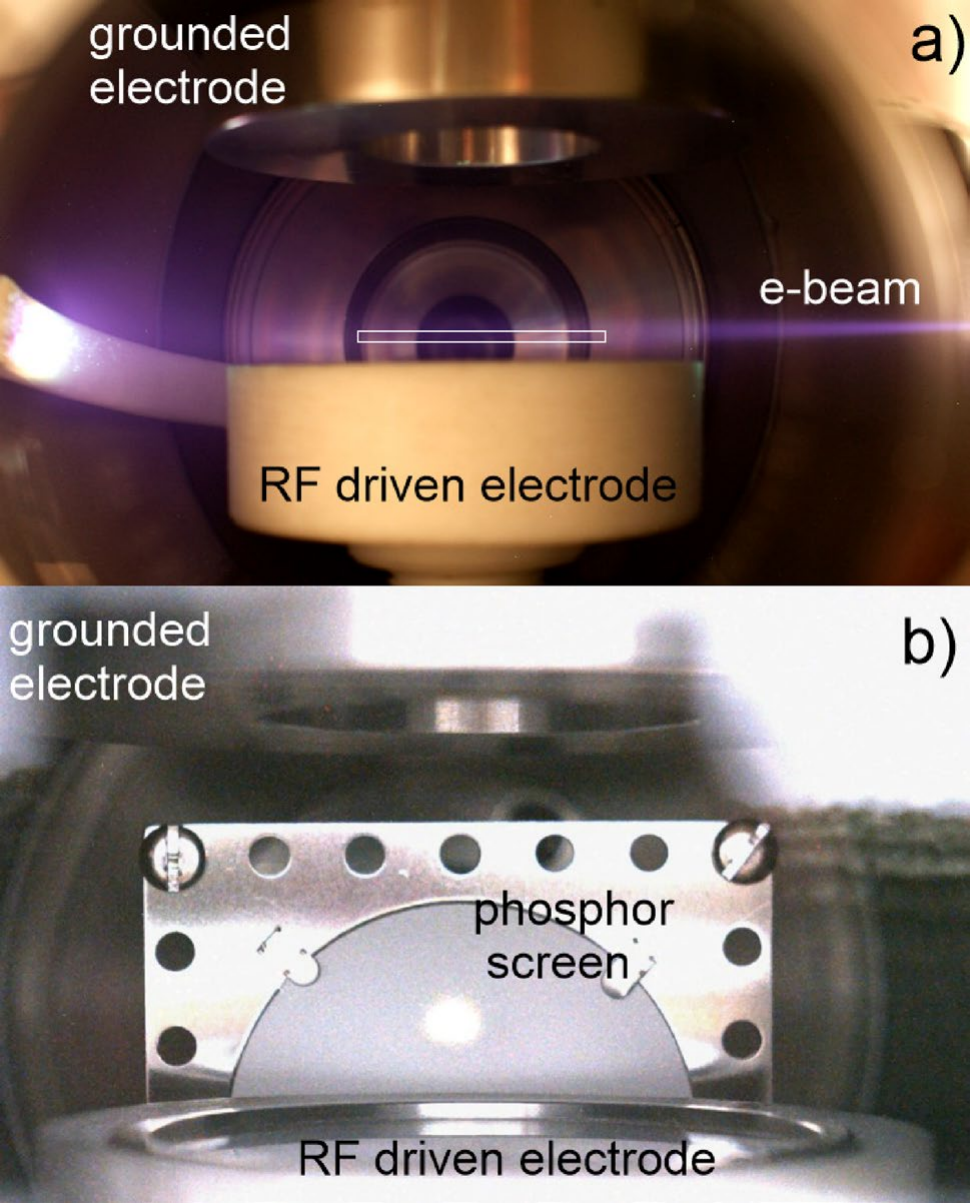
(**a**) Top grounded electrode and bottom electrode (insulated with Teflon which adds an extra 10 mm to the total size) driven by the RF signal inside the RF plasma chamber. The e-beam enters the chamber filled with Ar at 100 mTorr and passes between the electrodes, where the PC (not shown) is levitated. The thin rectangle indicates the levitation position of the dust crystal. On the left hand side the arm of the dust dropper made of Teflon blocks the e-beam and becomes fluorescent when irradiated; (**b**) Axial view along the e-beam direction: a phosphor screen is placed near the RF driven electron and used to image the e-beam for adjusting its height relative to the surface of the electrode.

The entering e-beam is aimed at the levitated crystal produced in a capacitive coupled RF plasma between two parallel-plate electrodes, as shown in Fig. 5a. The microparticles forming the crystal are illuminated with a red laser beam with a power of 20 mW (at $\lambda =680$ nm) and are imaged with a Photron high speed CCD camera through horizontal or vertical viewports. The lower electrode is a 50 mm in diameter disk provided with a shallow round cut of 1 mm height as shown in Fig. 5a, connected through a matching network to a RF power supply which delivers a high voltage at 13.56 MHz. The range of RF powers that can be fed to the electrode is between 1 and 100 W. The top electrode with a diameter of 71 mm is ring shaped and grounded. Dust particles are released into the RF plasma through the cut (with 30 mm in diameter) in the top electrode using a dropper and form a plasma crystal in the sheath of the bottom RF driven electrode. Spherical or cylindrical dust particles made of melamine formaldehyde (MF) or other materials such as silica, PMMA, etc. can be used. In the case of monodisperse dust spheres the diameter can range from submicron level to tens of microns, having a small standard deviation from about 0.04 to $0.14~\upmu$m for the smallest and largest spheres, respectively. The RF plasma is produced in argon at pressures between 50 and 200 mTorr.

The shallow cut in the bottom electrode has the role to confine the dust particles which are free to move in the horizontal plane, inside the plasma sheath. Near the cut the plasma sheath is curved and the electric field has a slight horizontal component pointing towards the center of the electrode. A radial electric force will be exerted on the nearby dust particles keeping them inside the area above the central part of the electrode. The height of the cut was chosen to be small enough (smaller than the levitation height) to allow the electron beam to reach the PC and also to allow the visualization of the particles from the side.

Vacuum in the full system of connected chambers is realized by a pumping system attached to the e-beam channel. It consists of a dry scroll pump running at 5.4 m$^3$h$^{-1}$ coupled to a turbomolecular pump with a pumping speed 250 L$\textrm{s}^{-1}$. The pumping system ensures the appropriate pressure regime for the simultaneous production of a collimated e-beam in high vacuum and of a PC at a much higher pressure in a separate chamber.

In order to collide the e-beam with the levitated PC an alignment procedure was put in place which uses a high luminosity screen for imaging the e-beam. A phosphor (ZnS:Ag Type 1330 - P22 blue) detector was placed near the RF driven electrode as shown in Fig. 5b. The e-beam produced a bright spot on the screen with a diameter of about 5 mm. After the screen was removed, the e-beam crossed over the full extent of the RF electrode at a few millimetres height which coincides with the levitation position of the dust cloud. The axis of the e-beam was aligned with the center of the RF driven electrode. For the uneven irradiation of a PC, e.g. the exposure of the side region of a PC, we could use a shallow round cut slightly asymmetric relative to the center of the electrode which confined the PC at a an off-axis location.

## Platform operation and diagnostics

**Figure 6:**
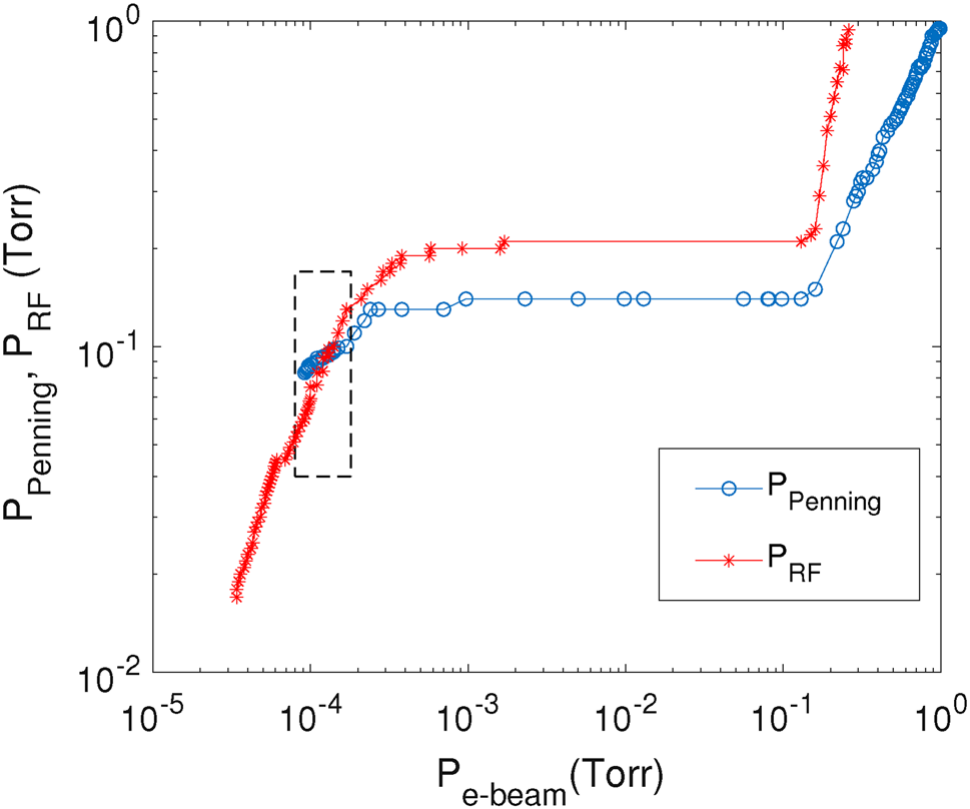
Pressures inside the Penning source $P_{Penning}$ and RF plasma chamber $P_{RF}$ versus the pressure inside the e-beam channel $P_{e-beam}$.The optimum working regime is marked by the rectangle.

One of the main challenges of this experimental set-up is to produce simultaneously both a stable e-beam accelerated at $\sim$ 8–14 kV and a PC taking into account that the gas pressures in the Penning discharge, the e-beam channel and the RF plasma chamber differ by several orders of magnitude. This can be achieved by setting appropriate pressure values in the vacuum chambers that communicate through small orifices. The optimum working regime is marked with a rectangle, as shown in Fig. 6.

In the initial operating step the turbo pump was used to pump down the whole system of vacuum chambers. In the second step, the e-beam was generated and then the crystal was produced in the RF plasma. The density $n_e$ and temperature $T_e$ of electrons in the RF plasma were measured with a commercially available Langmuir probe (Impendans Ltd.). The probe was compensated for the 13.56 MHz frequency and its first and second harmonics. Depending on the RF power supplied into the discharge, the measured values were in the range $n_e\approx 1\times 10^{14}-6\times 10^{14}$ m$^{-3}$ and $T_e\approx 3$–4 eV for RF powers from 1 to 5 W, respectively.

The e-beam pulses were acquired and their temporal profile was resolved. The current is a measure of the electrical charge carried by the e-beam. The standard way to measure the beam current is based on a Faraday cup (FC)^52^. A Kimball Physics model FC-72A has been used in our experiment. It was placed inside the RF chamber, next to the RF electrode. The exact position of the entrance aperture of the FC corresponded approximately to the edge of the RF electrode. The aperture diameter of the FC was 11.3 mm, about twice larger than the e-beam diameter. The signal of the FC was send through a BNC air/vacuum interface to a scope with 50 Ohms impedance while the waveform of the e-beam pulse could be monitored in real time.

**Figure 7:**
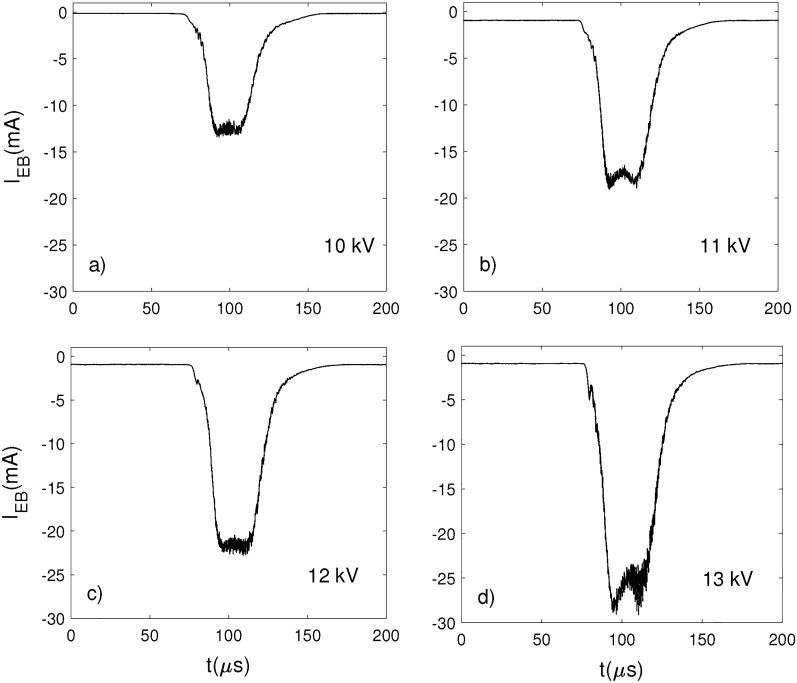
The beam current measured with the FC near the edge of the RF electrode for different accelerating voltages: (**a**) 10 kV, (**b**) 11 kV, (**c**) 12 kV and (**d**) 13 kV, in the RF vacuum chamber at a pressure $3.5\times 10^{-4}$ Torr. The pulse frequency was 46 Hz.

**Figure 8:**
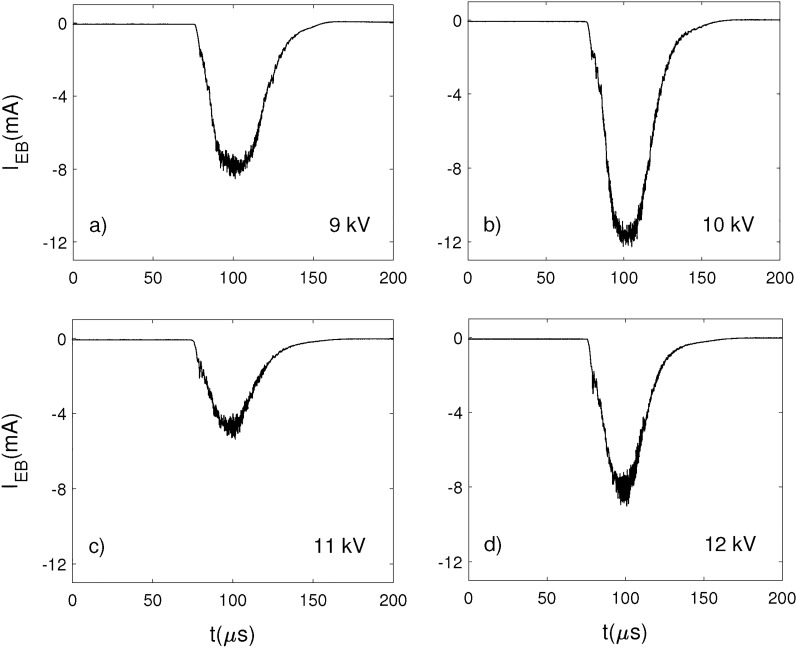
The beam current measured with the FC near the edge of the RF electrode for different accelerating voltages: (**a**) 9 kV, (**b**) 10 kV, (**c**) 11 kV and (**d**) 12 kV, in the RF vacuum chamber at a pressure $1.6\times 10^{-2}$ Torr. The pulse frequency was 93 Hz.

The measured e-beam pulses are presented in Figs. 7 and 8, with only one selected pulse shown. In Figs. 7a–c the e-beam pulse waveforms correspond to 10, 11, 12 and 13 kV accelerating voltages, respectively. These were obtained for particular magnetic fields configurations inside the two coils FC1 and FC2 biased with variable voltages. Thus, the waveform at 10 kV was obtained for $B_{FC1}=29$ G and $B_{FC2}=126$ G. The waveforms at 11 and 12 kV were obtained for relatively constant magnetic field in the first coil and slightly higher magnetic field in the second coil: $B_{FC1}=25$ G, $B_{FC2}=136$ G and $B_{FC1}=27$ G, $B_{FC2}=145$ G, respectively. In the case of 13 kV, the higher energy electrons needed a higher magnetic field in the first coil in order to get confined: $B_{FC1}=65$ G and $B_{FC2}=145$ G. The pulse duration of the e-beam at FWHM was about $40~\upmu$s. For all four cases the peak current was between 13 and 29 mA. The pulse repetition frequency was set at 46 Hz while the gas pressure in the RF chamber was $3.5\times 10^{-4}$ Torr.

In Figs. 8a–c a second set of pulsed e-beam waveforms are shown, but produced at a higher repetition frequency of 93 Hz. The gas pressure in the RF vacuum chamber was $1.6\times 10^{-2}$ Torr, in this case. The pulse duration was the same as in the previous measurements, however, the amplitude was lower, the maximum peak current value reaching 12 mA at 10 kV. The magnetic field had relatively close values for the 9 and 10 kV pulses shown in Figs. 8a,b: $B_{FC1}=15$ G, and $B_{FC2}=124$ G and $B_{FC1}=18$ G, $B_{FC2}=134$ G, respectively. In the case of 11 and 12 kV pulses a higher magnetic field was needed in the first coil in order to obtain stable e-beams: $B_{FC1}=67$ G, $B_{FC2}=128$ G and $B_{FC1}=63$ G, $B_{FC2}=136$ G, respectively.

## Rotational dust flow applications

### E-beam induced dust flow vortices

In previous works we have shown for the first time that an e-beam incident upon a PC can induce a dust flow in the crystal by the drag fore of the electrons acting on the individual dust particles. The flow speed vectors have been mapped in 2D and their evolution in space and time has led to the simultaneous formation of multiple small speed vortices^20,21^. The dust flow was dissipated towards the spatial limit of the crystal. The drag force exerted by the e-beam was sufficient to imprint an acceleration up to a terminal speed of the order of a few mms^−1^. The main opposing forces were the friction with the neutral gas and the Coulomb repulsion exerted by the neighboring particles. In those cases the e-beam current was as small as $\sim 4$ mA. The entrainment of the dust particles by the e-beam led to a local disorder in the crystal involving dust-dust collisions. This effect coupled with the stochastic charging of dust in plasma, partially influenced by the injection of new electrons from the e-beam, forced the dust particles to move on more random trajectories which eventually formed vortices along the flow direction.

The keV e-beam induces a negligible charging on the large dust particles (with diameters $\sim 10~\upmu$m) by secondary electron emission (SEE), or from scattered electrons by neighboring particles, which further get thermalized and are trapped on the surface. The SEE current is of order $10^{-11}-10^{-10}$ A, while the electron charging current drawn from the plasma is at least one order of magnitude higher ($\approx 10^{-9}$ A)^21^. Taking into account that the charging time of the microparticles in plasma is of order of a few $\upmu$s, the e-beam pulse has a duration of 40 $\upmu$s and the period between pulses is tens of ms, the charge variation induced by SEE and by scattering of electrons is restored rapidly to the equilibrium value set by the plasma currents such that the microparticles do not modify their levitation height above the lower electrode^21^. It is, however, of interest to study the interaction of the e-beam also with small dust particles with diameters $\lesssim$ 2–3 $\upmu$m levitated in plasma. In this case the SEE can play a more important role in the particle charging process. At the same time it is expected that the energetic electrons from the beam will pass through the dust particles exiting on their opposite side.

**Figure 9:**
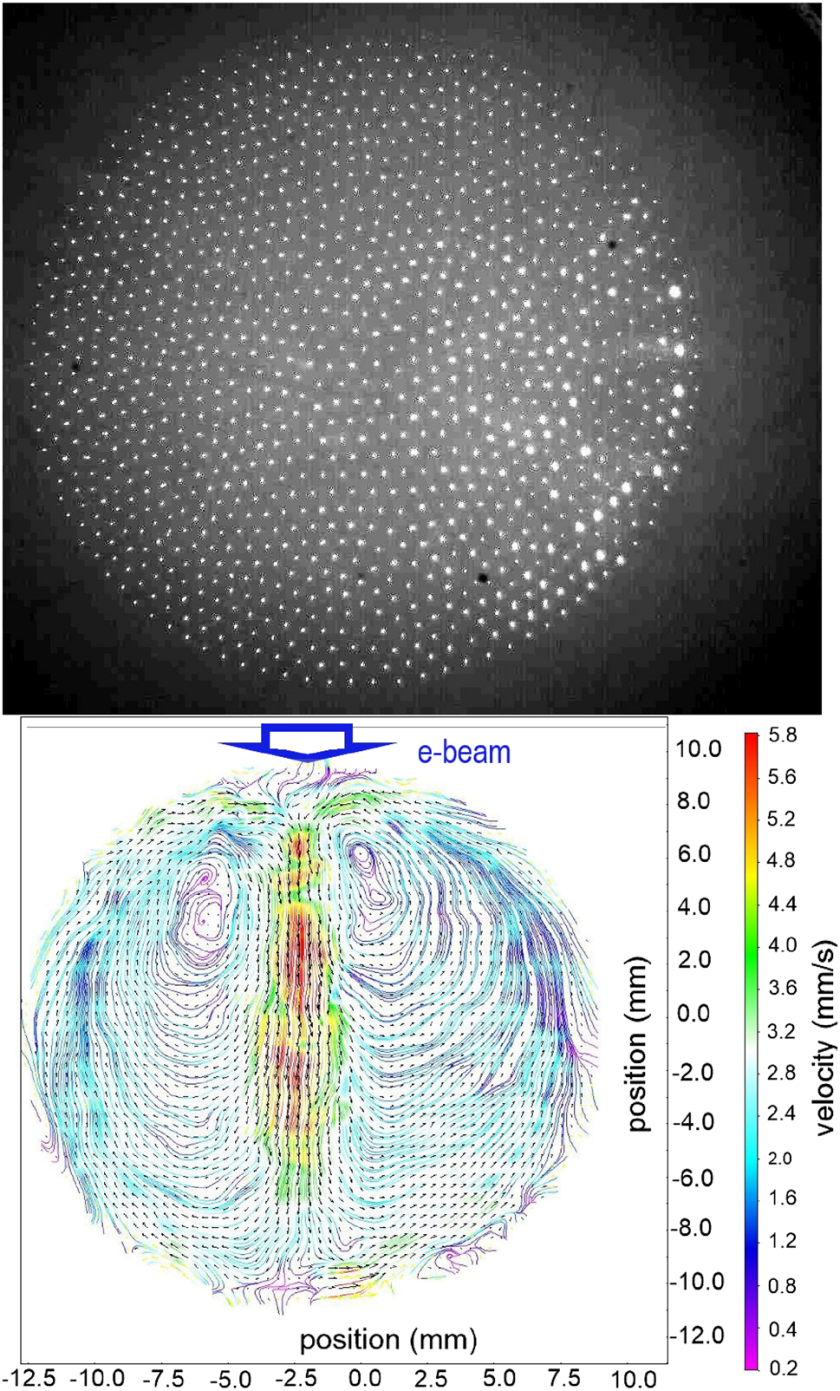
(**a**) Top view of a large quasi-2D PC (**b**) PIV image of the e-beam induced dust flow forming two symmetrical vortices relative to the irradiation direction. The streamlines show the geometry of the flow while the flow speed is inferred from the color bar (see supplementary movie file with the dust flow vortices).

We demonstrate here that a more intense e-beam with a peak current of 30 mA can induce a much longer laminar dust flow, over the entire section of a PC as shown in Fig. 9. The electrons in the beam were accelerated at 13 kV and the pulse frequency was 93 Hz. The top view of a quasi-2D crystal is shown in Fig. 9a. The crystal was 2D (i.e. having a single layer of dust particles) except for a small number of randomly trapped dust particles underneath the top layer, which did not influence the observations. Dust particles made of MF with a diameter of $11.8~\upmu$m were used. One can see the formation of two large symmetrical vortices on both sides of the irradiation axis, which basically split the crystal in two, as shown in Fig. 9b. The image given by particle image velocimetry (PIV) shows the flow direction while the streamlines indicate the closed trajectories of the entrained dust particles. The top speed of the dust flow was measured along the e-beam direction and reached 5.8 mms$^{-1}$. We found that the dust flow properties such as speed and structure were dependent on the e-beam parameters (current, acceleration voltage, width and direction).

Our experimental platform is versatile in the sense that we have the capability to tune the e-beam parameters on one hand, but we can also modify the properties of the PC on the other hand. We can adjust the e-beam current, its energy and even its cross section (e.g., by varying the inlet hole of the e-beam in the PC chamber). We can also modify the plasma electron density and the neutral gas density, along with the electric field profile inside the plasma sheath where the crystal resides. Moreover, we can always use different types of dust particles with different mass density (given by different materials) and having mono-disperse or poli-disperse sizes.

A PC can undergo a phase transition from solid-like to liquid-like state under the action of an external constraint such as the reduction of gas pressure, leading to a reduced friction with the neutral gas^53^. During the melting process the Coulomb coupling parameter $\Gamma$ decreases from $\Gamma \sim 10^3$ for a strongly coupled crystal to $\Gamma \lesssim$ 100–200 for the melted crystal. In our case the crystal can melt locally when irradiated by the e-beam. It has been demonstrated that the charged dust fluid is non-Newtonian, featuring shear thinning particularly at low shear rates, with its viscosity varying strongly with $\Gamma$^54^. Low shear rates can be attained at lower e-beam currents, e.g. at 4 mA when we observe the formation of multiple vortices at different space scales and with a wide range of vorticities^20^. Tuning the viscosity of the charged dust flow means that its Reynolds number can be modified, which is one key asset of our platform. This allows direct access to study the onset of turbulence in experiments with charged dust fluids, which is of course influenced by many parameters.

**Figure 10:**
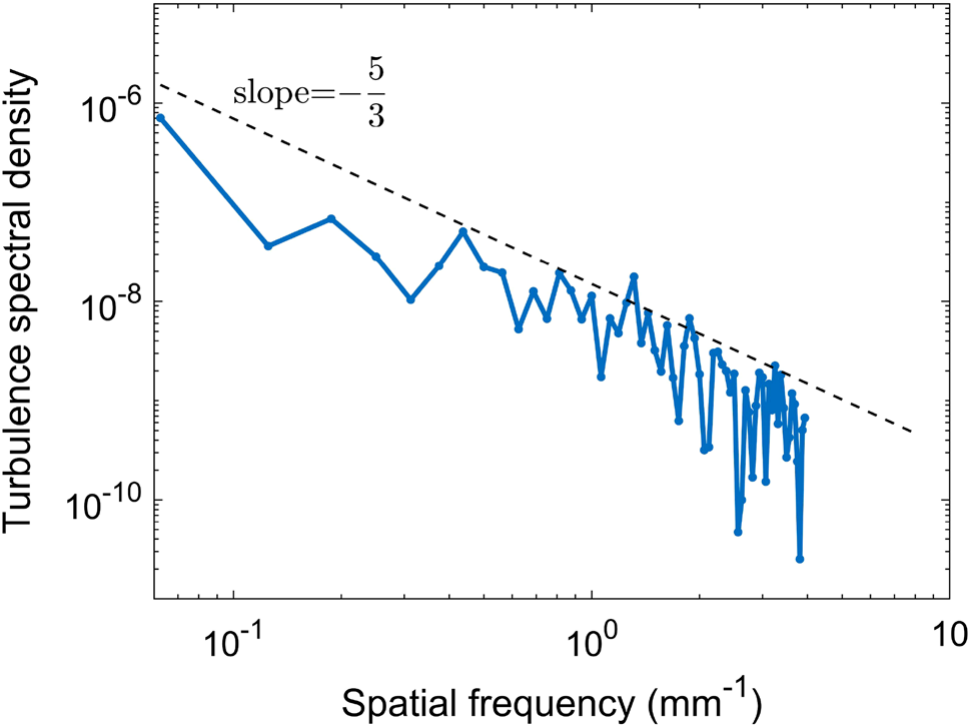
Turbulence energy spectrum of a dust flow induced by an e-beam in a quasi 2D plasma crystal. The dashed line indicates the theoretical $-\frac{5}{3}$ slope of the inertial range (see supplementary movie file with the turbulent dust flow).

The charged dust flow behaves as a viscoelastic fluid, with a more pronounced elastic character at a small scale length (of the order of the inter-particle distance, i.e. a few hundred microns) and with a more viscous character at a large scale^55^. Turbulence in viscoelastic fluids has been shown to arise at much lower Reynolds numbers than in Newtonian ones^56^. This is the case of our dust flow composed of monodisperse charged dust particles with a diameter of 11.8 $\upmu$m pushed by the 4 mA and 13 keV e-beam, at a few Watts of RF power and at 84 mtorr neutral gas pressure: the Reynolds number is $\textrm{Re}\simeq 50$^20^. This dust flow is turbulent featuring vortices with large vorticities, in the range $\approx -10...10$ s$^{-1}$, that are constantly created and dissipated along its length. An analysis of the spectral turbulent energy of the flow using the 2D speed vectors obtained by PIV in the plane of the crystal, along the flow axis and at a specific moment in time, is presented in Fig. 10. One can clearly see that the spectrum features an inertial range well fitted by the Kolmogorov power law with slope $-5/3$^57^.

### E-beam induced rotation of a plasma crystal

**Figure 11:**
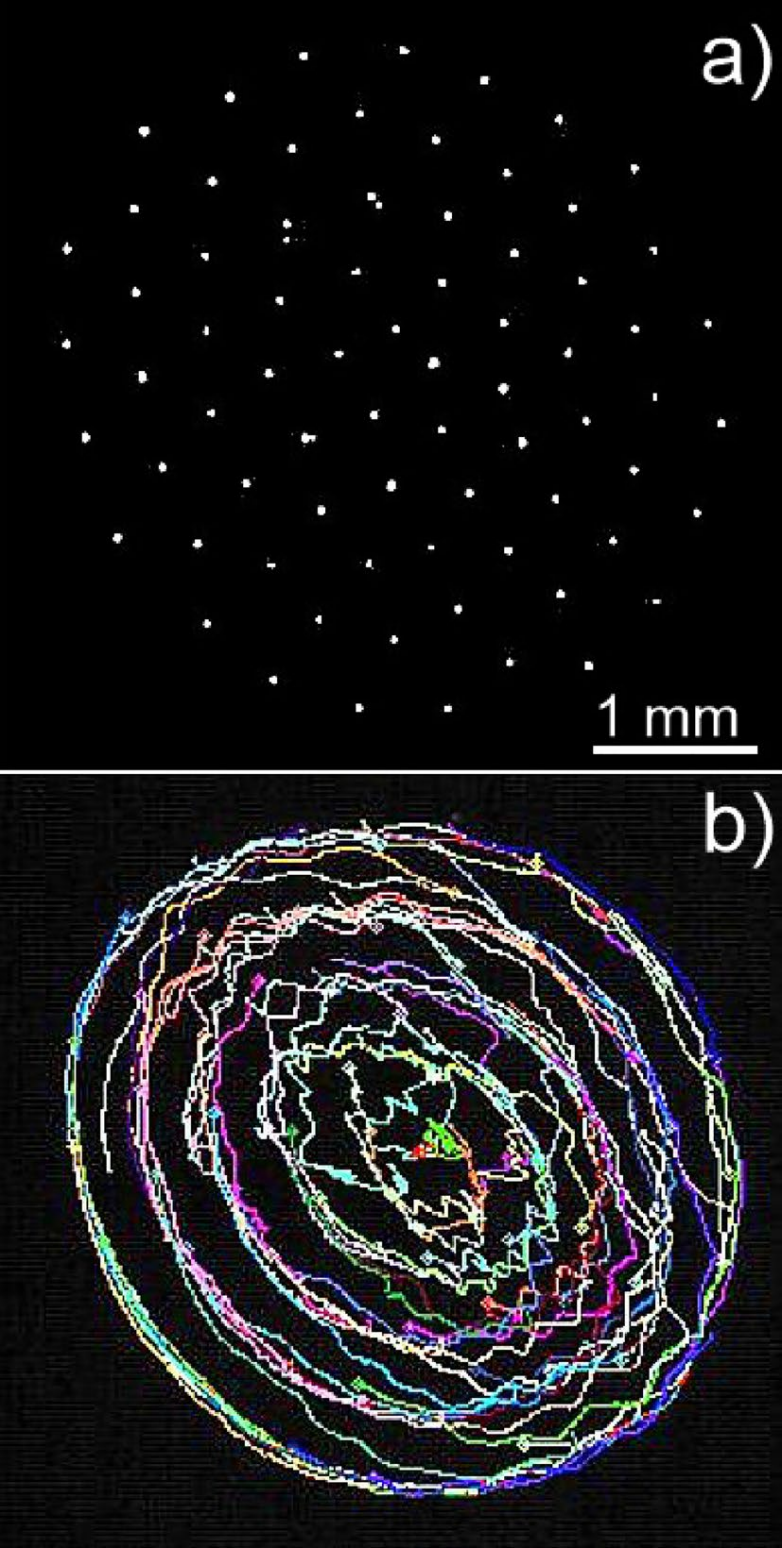
(**a**) Top view of a quasi 2-D PC (**b**) e-beam induced rotation of the plasma crystal and dust particles trajectories obtained using the PTV technique. Each dust particle trajectory has a different color (see supplementary movie file with the crystal rotation).

A quasi 2D crystal made of similar dust particles as in the previously presented experiment was irradiated with the e-beam which was slightly misaligned relative to the center of the crystal, in a similar fashion presented in ref.^30^. Thus, a side of the crystal was more exposed to the e-beam than the rest of it with the e-beam impinging on the irradiated dust particles positioned at the edge. Here the e-beam was accelerated at 14 kV, the beam current was 4.5 mA and the frequency was 46 Hz. The dust particles confined by the RF sheath potential were kept together by their strongly coupling force, resulting in the generation of a torque acting on the whole PC. The net results was a rotation of the crystal in the direction of the e-beam, as presented in Fig. 11. The top view of the PC is shown in Fig. 11a. The trajectories of the dust particles composing the irradiated crystal are shown in Fig. 11b obtained by employing the particle tracking velocimetry (PTV) technique available on a dedicated software package^58,59^. The concentring rings show that the structure of the crystal was broadly preserved, except for some jumps as portrayed by the crossing lines between the concentring rings. A few dust particles positioned near the center of the crystal followed some discrete jumps and exchange positions, rotating further at their new locations without destroying the symmetry of the crystal. The speed of the particles varied from $\approx 0.2$ mm$\textrm{s}^{-1}$ for the inner dust particles close the crystal center to $\approx 0.6$ mm$\textrm{s}^{-1}$ for the edge dust particles. The average inter-particle distance was 0.52 mm, resulting in an angular speed of rotation $0.25\pm 0.05$ rad$\textrm{s}^{-1}$.

## Conclusions

We presented the main features of a new laboratory platform dedicated to studying the interaction of e-beams with strongly-coupled dust particles levitated in plasma. The platform combines several elements in a unitary way, such as the extraction of free electrons from a Penning discharge, their subsequent acceleration at about 10 kV, the collimation of these electrons into an e-beam and the irradiation of a PC formed in a RF discharge. We demonstrated the capability of the platform by presenting some novel results concerned with the formation of dust flow vortices inside a PC as a result of the drag force exerted on the dust particles by the e-beam. Also we demonstrated that a PC is rotated by the uneven exposure to the circular e-beam due to the creation of a torque that pushes the side dust particles faster. The platform is especially suited to study the unique dynamical properties of charged Coulomb-coupled flows, the transition from their laminar to turbulent states and the formation of dust vortices. Other new phenomena will be studied as well such as the unusual sudden acceleration of only a few dust particles to high speeds, larger than the dust flow speed, most likely related to the extra-charging of the dust particles by the e-beam, or the Coulomb explosion of irradiated dust clusters.

## Supplementary Information

Supplementary Video 1.

Supplementary Video 2.

Supplementary Video 3.

## Data Availability

All data generated or analysed during this study are included in this published article and its supplementary information files which contain 3 video files, one with the rotating plasma crystal, the second with the double vortex formed in the dust flow and the third showing the turbulent dust flow.
